# Regulation of Endogenous Glucose Production in Glucose Transporter 4 Over-Expressing Mice

**DOI:** 10.1371/journal.pone.0052355

**Published:** 2012-12-17

**Authors:** Eric D. Berglund, Candice Y. Li, Julio E. Ayala, Owen P. McGuinness, David H. Wasserman

**Affiliations:** 1 Department of Molecular Physiology and Biophysics, Vanderbilt University School of Medicine, Nashville, Tennessee, United States of America; 2 Mouse Metabolic Phenotyping Center, Vanderbilt University School of Medicine, Nashville, Tennessee, United States of America; National Institutes of Health, United States of America

## Abstract

Strategies to amplify whole-body glucose disposal are key therapies to treat type 2 diabetes. Mice that over-express glucose transporter 4 (Glut4) in skeletal muscle, heart, and adipose tissue (G4Tg) exhibit increased fasting glucose disposal and thus lowered blood glucose. Intriguingly, G4Tg mice also exhibit improved insulin-stimulated suppression of endogenous glucose production even though Glut4 is not present in the liver. It is unclear, however, if hepatic gluco-regulation is altered in G4Tg mice in the basal, non-insulin-stimulated state. The current studies were performed to examine fasting hepatic glucose metabolism in G4Tg mice and to determine whether gluco-regulatory adaptations exist in the non-insulin-stimulated condition. To test this question, phloridzin-glucose clamps were used to match blood glucose and pancreatic hormone levels while tracer dilution techniques were used to measure glucose flux. These techniques were performed in chronically-catheterized, conscious, and un-stressed 5h-fasted G4Tg and wild-type (WT) littermates. Results show reduced blood glucose, hepatic glycogen content, and hepatic glucokinase (GK) activity/expression as well as higher endogenous glucose production, glucose disposal, arterial glucagon, and hepatic glucose-6-phosphatase (G6Pase) activity/expression in G4Tg mice versus WT controls. Clamping blood glucose for 90 min at ∼115 mg/dLin G4Tg and WT mice normalized nearly all variables. Notably, however, net hepatic glycogen synthetic rates were disproportionately elevated compared to changes in blood glucose. In conclusion, these studies demonstrate that basal improvements in glucose tolerance due to increased uptake in extra-hepatic sites provoke important gluco-regulatory adaptations in the liver. Although changes in blood glucose underlie the majority of these adaptations, net hepatic glycogen synthesis is sensitized. These data emphasize that anti-diabetic therapies that target skeletal muscle, heart, and/or adipose tissue likely positively impact the liver.

## Introduction

Defects in gluco-regulation define type 2 diabetes, and therapies to control dysregulated blood glucose are a fundamental component of disease management. A network of organs including skeletal muscle, heart, adipose tissue, and liver contribute to regulating whole-body glucose homeostasis [1]. Glucose transporter 4 (Glut4) is an insulin- and exercise-sensitive glucose transporter present in skeletal muscle, heart, and adipose tissue [1]–[3]. Over-expression of Glut4 in these sites results in a mouse model (G4Tg) characterized by lowered blood glucose, reduced insulin, and improved whole-body glucose tolerance [4]–[9]. G4Tg mice also exhibit improved whole-body insulin action as shown by increased exogenous glucose infusion requirements to maintain euglycemia during a hyperinsulinemic-euglycemic clamp [4], [8]. Surprisingly, this is not due to enhanced insulin-stimulated glucose uptake in short-term fast mice [4], [8]. Instead, insulin-mediated suppression of endogenous glucose production accounted for higher glucose requirements in G4Tg mice [4], [8]. These results suggest that G4Tg mice exhibit a hepatic phenotype. This is noteworthy because the liver does not express Glut4.

The present study tests the hypothesis that an increase in whole-body peripheral glucose disposal due to over-expression of Glut4 in skeletal muscle, heart, and adipose tissue provokes adaptations in basal hepatic glucose metabolism. Liver glucose production was assessed under fasting conditions as well as during phloridzin-glucose clamps. The latter was done to acutely abolish differences in blood glucose, and thus insulin and glucagon, between wild-type (WT) and G4Tg littermates. These analyses were performed using chronically-catheterized mice and isotopic tracer techniques which permit serial blood sampling and analysis of glucose flux in conscious, unstressed animals [10].

## Methods

### Animals Care and Husbandry

All procedures were approved by the Vanderbilt University Animal Care and Use Committee. G4Tg mice on a C57BL/6J background have been previously described [11]. Littermates were used as controls. Equal numbers of male and female mice were used in each group. Mice had free access to a standard chow diet and water and were studied at four months of age.

### Surgical Procedures

Surgical procedures to implant catheters in the left common carotid artery and right jugular vein for sampling and infusing, respectively, have been previously described [10], [12]. Mice were housed individually during a 5 d recovery. Only mice returning to within 10% of pre-surgical body weight were studied.

### Phloridzin-Glucose Clamp

On the day of the study, mice began a 5 h fast at ∼8:00am to ensure that animals are post-absorptive and replete with liver glycogen [10]. At t  = −90 min, [3-^3^H]glucose was infused (50 μCi bolus +0.05 μCi·min^−1^) to measure glucose turnover. Blood samples were taken at t = −30, −20, −10, and 0 min to determine basal endogenous glucose appearance (endoR_a_) and disappearance (R_d_). Blood samples were taken at t = −30 and t = 0 min to measure basal arterial glucagon and insulin. Additional blood was taken at t = −30 min to measure basal non-esterified fatty acids (NEFAs) and hematocrit. A continuous infusion of saline-washed erythrocytes was started at t = −30 min to prevent a >5% fall in hematocrit. At t = 0 min, phloridzin (80 μg·kg^−1^·min^−1^) was infused and the [3-^3^H]glucose infusion was increased to 0.1 μCi·min^−1^. Arterial blood glucose was measured every 10 min thereafter using a glucose meter. A variable glucose infusion rate (GIR) was adjusted to maintain euglycemia at ∼115 mg·dl^−1^. Blood samples were taken at t = 60, 70, 80 and 90 min to determine glucose turnover. Additional samples were taken at t = 60 and 90 min to assess insulin, glucagon, and NEFA. Blood was taken at t = 80 min to assess hematocrit. Mice were anesthetized at t = 90 min and tissues were excised and frozen. In a subgroup of animals (n = 7 mice/genotype), mice were anesthetized at t = 0 min (e.g. prior to infusion of phloridzin) and tissues were excised and frozen to provide basal data.

### Arterial Plasma Analyses

The measurement of [3-^3^H]glucose in plasma was performed as described [10]. Insulin and glucagon were determined by the Vanderbilt Mouse Metabolic Phenotyping Center (MMPC) [13]. NEFA were determined using a kit (Wako, NEFA C kit, Wako Chemicals Inc., Richmond VA).

### Tissue Analyses

Liver samples used to assess glucokinase (GK) and glucose-6-phosphatase (G6Pase) activity were homogenized 1∶10 (w/v) in buffer (50 mM HEPES, 0.1 mM KCl, 1 mM EDTA, 5 mM MgCl_2_ and 2.5 mM dithioerythritol) and centrifuged (100,000 g) at 4°C for 45 minutes. GK activity was assessed on supernatant. G6Pase activity was determined on pellets after re-suspension in buffer. For GK assays, supernatants were incubated at 37°C in buffer (50 mM HEPES, 0.1 mM KCl, 7.5 mM MgCl_2_, 2.5 mM dithioerythritol, 10 mg/ml NAD, 4 U/ml glucose-6-phosphate dehydrogenase, 5 mM ATP, and either 0.5 mM or 100 mM glucose). GK activity was calculated as the difference in radioactivity in the presence of 0.5 mM and 100 mM glucose. For G6Pase assays, samples were incubated at 37°C for 20 min with 0, 10, 20, 50, 100, or 200 mM glucose-6-phosphate.

Liver samples to assess glycogen phosphorylase (GP) and glycogen synthase (GS) were homogenized 1∶10 (w/v) in buffer (50 mM Tris (pH 6.8), 10 mM EDTA, 100 mM NaF, 5 mM dithioerythritol, and 0.5% glycogen). GP activity was measured after incubation of supernatant with phosphorylase reaction medium (150 mM NaF, 1.5% glycogen, 15 mM glucose-1-phosphate, 5 uCi [^14^C]glucose-1-phosphate, and either 0.75 mM caffeine or 7.5 mM AMP). GS activity was assessed following addition of supernatant to synthase reaction medium (50 mM Tris (pH 7.5), 5 mM EDTA, 1% glycogen, 1.5 mM UDP-glucose, 5 uCi [14C] UDP-glucose, and either 15 mM Na_2_SO_4_ or 3 mM glucose-6-phosphate). Reactions were incubated at 37°C and aliquots were spotted onto Whatman chromatography filter paper every 10 min for 40 min. Samples were washed in ethanol and radioactivity was measured. GP activity was calculated by the ratio of activity in the presence of 7.5 mM AMP compared to 0.75 mM caffeine. GS activity was calculated by the ratio of activity in the presence of 3 mM glucose-6-phosphate compared to 15 mM Na_2_SO_4_.

Liver glycogen was determined enzymatically as previously described [14]. Hepatic mRNA was determined as previously described [15]. Taqman Assay-on-Demand primers were used to measure expression of genes of interest normalized to cyclophilin using the ΔΔC_t_ calculation.

### Calculation and Statistical Analyses

EndoR_a_ and R_d_ were calculated as previously described [10]. All values are presented as means ± SE. Differences between groups were tested using Student's *t* tests and differences were considered significant if *p*<0.05.

## Results

**Figure 1:**
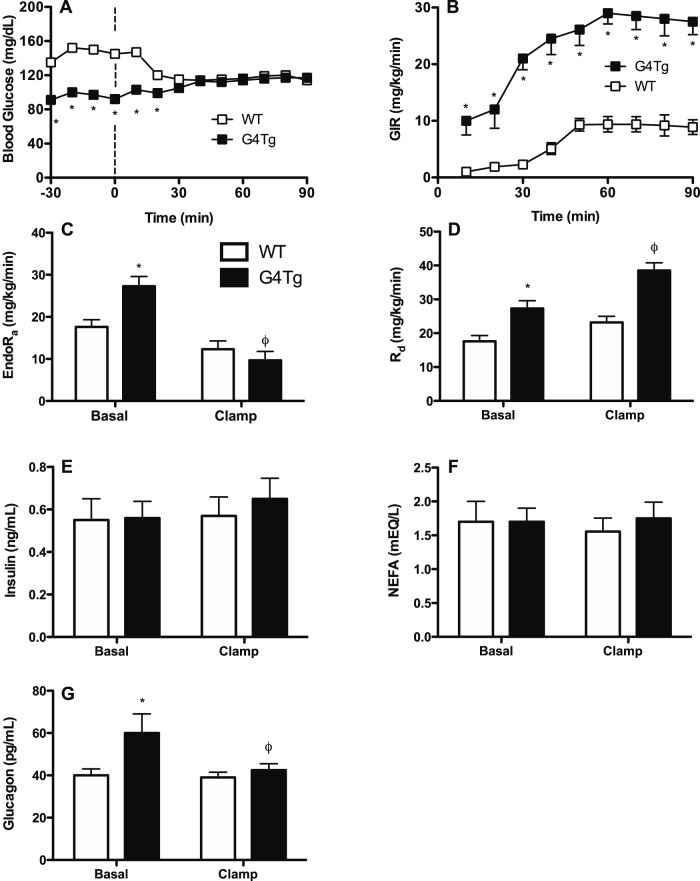
Hormonal and glucose flux responses to acutely normalizing blood glucose in glucose transporter 4 over-expressing mice (G4Tg) and wild-type (WT) littermates. A 90 min phloridzin (80 μg·kg^−1^·min^−1^)-glucose (115 mg·dL^−1^) clamp was performed in conscious, chronically-catheterized, 5 h-fasted mice that over-express glucose transporter 4 (Glut4) in skeletal muscle, heart, and adipose tissue and wild-type (WT) littermates to normalize basal differences in arterial blood glucose (A) using an exogenous glucose infusion rate (B). Basal and clamp endogenous appearance (endoR_a_; C) and disappearance (R_d_; D) of glucose were measured using a primed, constant infusion of [3-^3^H] glucose (50 μCi bolus +0.05 μCi·min^−1^). Basal and clamp arterial insulin, non-esterified fatty acids (NEFA), and glucagon are shown in panels E-G, respectively. Data are presented as means ± SEM and * and ϕ indicate p<0.05 compared to WT littermates or to basal values within a genotype, respectively. n = 7–8 mice in each group.

Phloridzin-glucose clamps were performed in G4Tg and WT littermate mice to normalize basal 5 h fasted differences in arterial blood glucose, insulin, glucagon, and glucose turnover (Table 1 and Figure 1A). There were no differences in body weight between the two genotypes (Table 1). During the clamp, the GIR required to achieve the target arterial glucose concentration of ∼115 mg/dL (Figure 1A) was ∼3-fold higher in G4Tg mice (Figure 1B). Relative changes in blood glucose levels from basal to clamp steady-state conditions were comparable (+16.9±1.8 and −17.5±1.8% from basal in G4Tg and WT mice, respectively).

**Table 1:** Basal characteristics of wild-type (WT) and littermate mice with over-expression of glucose transporter 4 (G4Tg).

	WT	G4Tg
*n*	16	14
Body Weight (g)	24±1	26±1
Blood Glucose (mg·dL^−1^)	141±8	91±3*
Insulin (ng·mL^−1^)	0.6±0.1	0.6±0.1
Glucagon (pg·mL^−1^)	41±4	60±10*
NEFA (mEqL^−1^)	1.7±0.3	1.7±0.2
EndoR_a_ (mg·kg·min^−1^)	17±1	26±2*
R_d_ (mg·kg·min^−1^)	17±1	26±2*

Measurements were taken in 5h fasted mice prior to phloridzin-glucose clamps and represent combined data from control and experimental animals. * indicates p<0.05 compared to WT littermates.

### Glucose Kinetics

Basal endoR_a_ and R_d_ were higher in G4Tg mice versus WT littermates (Figures 1C and 1D). In response to equalizing blood glucose, clamp endoR_a_ was lowered in G4Tg mice to rates comparable to WT mice (Figure 1C). Clamp endoR_a_ in WT mice remained similar to basal rates (Figure 1C). Clamp R_d_ in G4Tg mice was elevated from basal rates and was higher than WT mice (Figure 1D). Clamp R_d_ was unchanged from basal rates in WT mice (Figure 1D).

### Arterial Insulin, Glucagon, and NEFA Concentrations

Basal arterial insulin and NEFA levels were comparable in G4Tg and WT littermates and were unaltered by clamp-induced normalization of arterial glucose (Figures 1E and 1F). Basal arterial glucagon levels in G4Tg mice were higher than WT littermates (Figure 1G). This difference was abolished by normalization of arterial glucose during the clamp (Figure 1G).

### Changes in Hepatic Glycogen, Enzyme Activity, and Gene Expression

**Figure 2:**
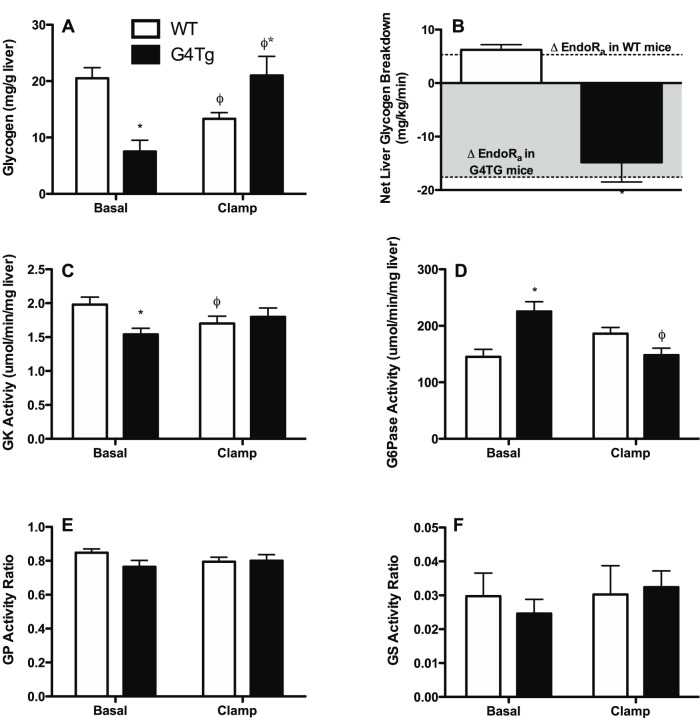
Hepatic glycogen and related enzyme activities in response to acutely normalizing blood glucose in glucose transporter 4 over-expressing mice (G4Tg) and wild-type (WT) littermates. Hepatic glycogen content (A), hepatic glycogen breakdown (B), and activities or activity ratios of glucokinase (GK; C), glucose-6-phosphatase (G6Pase; D), glycogen phosphorylase (GP; E), and glycogen synthase (GS; F)] are shown before and after normalizing blood glucose in 5 h-fasted mice that over-express glucose transporter 4 (Glut4) in skeletal muscle, heart, and adipose tissue and wild-type (WT) littermates using a 90 min phloridzin (80 μg·kg^−1^·min^−1^)-glucose (115 mg·dL^−1^) clamp. Separate cohorts of mice were used to obtain basal and clamp data. Dashed lines in panel B denote changes in endogenous appearance of glucose (endoR_a_; shown in Figure 1C). Data are presented as means ± SEM and * and ϕ indicate p<0.05 compared to WT littermates or to basal values within a genotype, respectively. n = 7–8 mice in each group.

Basal 5 h fasted hepatic glycogen content was lower in G4Tg mice versus WT littermates (Figure 2A). WT mice had an ∼25% reduction in hepatic glycogen over the course of the experimental period (Figure 2A). In contrast, normalizing arterial glucose by the phloridzin-glucose clamp resulted in repletion of hepatic glycogen levels in G4Tg mice to those observed in WT mice in the basal state (Figure 2A). Changes in liver glycogen (shown as net hepatic glycogen breakdown in Figure 2B) associated with normalizing arterial glucose are nearly equal to the respective changes in endoR_a_ in G4TG and WT littermate mice (denoted by dashed lines in Figure 2B and shown in Figure 1C).

**Figure 3:**
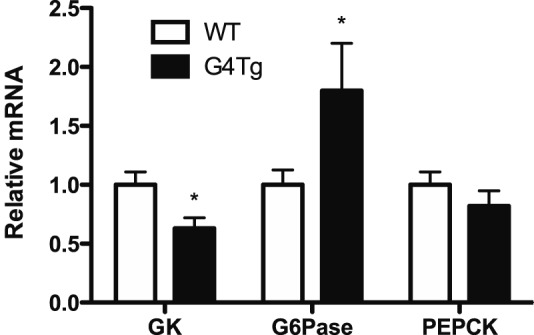
Hepatic expression of gluco-regulatory enzymes after acutely normalizing blood glucose in glucose transporter 4 over-expressing mice (G4Tg) and wild-type (WT) littermates. Hepatic expression of glucokinase (GK; A), glucose-6-phosphatase (G6Pase; B), and cytosolic phospho*enol*pyruvate kinase (PEPCK; C) are shown before and after normalizing blood glucose in 5h-fasted mice that over-express glucose transporter 4 (Glut4) in skeletal muscle, heart, and adipose tissue (G4Tg) and wild-type (WT) littermates during a 90 min phloridzin (80 μg·kg^−1^·min^−1^)-glucose (115 mg·dL^−1^) clamp. Separate cohorts of mice were used to obtain basal and clamp data. Data are presented as means ± SEM and normalized to cyclophilin expression and basal levels in WT mice. * and ϕ indicate p<0.05 compared to WT littermates or to basal values within a genotype, respectively. n = 7–8 mice in each group.

To better understand differences in glycogen metabolism and glucose fluxes, activities and expression of key gluco-regulatory enzymes were measured in the basal state and following the clamp in G4Tg and WT littermates. Consistent with the accelerated rate of hepatic glucose production, basal hepatic GK and G6Pase enzyme activities were lower and higher, respectively, in G4Tg mice compared to WT controls (Figures 2C and 2D). Normalizing blood glucose between the two genotypes eliminated these differences (Figures 2C and 2D). In contrast to GK and G6Pase, hepatic GP and GS enzyme activity ratios did not differ between G4Tg mice and WT littermates and were unchanged following the clamp (Figures 2E and 2F). In agreement with hepatic enzyme activity ratio data, basal hepatic expression of GK and G6Pase were lower and higher, respectively, in G4Tg mice versus WT littermates (Figures 3A and 3B). Hepatic GK expression was elevated in both genotypes to a similar extent following the clamp (Figure 3A). Hepatic G6Pase gene expression was increased and decreased with respect to expression in the basal state in WT and G4Tg mice, respectively (Figure 3B).

## Discussion

These current data in G4Tg mice prior to normalizing blood glucose are consistent with previous studies showing lowered blood glucose, higher glucagon, and comparable NEFA levels compared to WT littermates [4], [6]–[9]. The present results also support previous work demonstrating enhanced whole-body glucose production and glucose disposal in G4Tg mice [8]. The current findings in the basal, pre-clamp condition provide additional details that G4Tg mice exhibit an altered hepatic gluco-regulatory phenotype that is characterized by higher and lower activities and gene expression of G6Pase and GK, respectively, as well as greatly reduced liver glycogen content. This shift in carbohydrate flux away from storage in the liver is consistent with the need to support elevated systemic glucose removal from the blood in the post-absorptive state.

The most salient findings from this work were obtained using phloridzin-glucose clamps to study G4Tg mice in the absence of hyperinsulinemia or differences in arterial glucose levels compared to WT littermates. Phloridzin is an agonist for sodium/glucose co-transporters in kidney and intestine and is used in the current context as a tool in conjunction with an exogenous glucose infusion to establish a comparable glycemic background [16]. Importantly, these glucose clamp results reveal that acutely creating a comparable glycemic background eliminates many, but not all differences observed in the basal (e.g. pre-clamp) condition. Notably, matching arterial blood glucose to the mid-point of the respective 5 h fasted levels in G4Tg and WT littermate mice results in comparable clamp endoR_a_ and plasma glucagon levels. Clamp results in G4Tg mice do suggest a disproportionate increase in hepatic glycogen content relative to the change in blood glucose when compared to WT littermates. This conclusion is based on the fact that the magnitude of change in blood glucose was similar between G4Tg and WT mice, but the corresponding change in hepatic glycogen content was greater in G4Tg mice. In addition, hepatic GK expression does not fall in G4Tg mice despite the normalization of blood glucose. These findings suggest that the G4Tg mouse liver is primed to store liver glycogen.

Although the purpose of the present experiments was not to study changes in blood glucose *per se*, the clamp results in both genotypes also demonstrate how sensitive hepatic gluco-regulatory pathways are to changes in blood glucose. In particular, these data demonstrate that modest and acute changes in blood glucose potently alter hepatic GK and G6Pase expression in both G4Tg and WT mice. It is important to consider that these aforementioned changes occur after only a 60 min steady-state period during which arterial glucose was matched to the same intermediate value of ∼115 mg/dL. It is also important to note that this approach to use the mid-point between the two genotypes as the glucose clamp target instead of the more extreme approach of raising or lowering the respective blood glucose levels in G4Tg or WT to match the other genotype was selected because it allowed steady-state conditions to be obtained in both genotypes in a shorter time frame.

An additional experimental point of emphasis in the current studies is that blood glucose was controlled in WT and G4Tg using a clamp protocol in which the jugular vein and carotid artery were chronically catheterized for sampling and infusing purposes, respectively. This mouse model differs from those generally used [8] and is advantageous because it does not induce a stress response (e.g. a rise in catecholamines) that occurs with the more common tube restraint and cut-tail blood sampling approach [10]. This point is important in the current context because increases in stress would be predicted to alter both hepatic and extra-hepatic glucose metabolism [10], [17]. Erythrocytes were also replaced in the current experiments to prevent the fall in hematocrit that occurs following repeated blood sampling and consequent loss of red blood cell volume.

In conclusion, these experiments show that a peripheral increase in fasting glucose disposal exerts positive effects on liver glucose metabolism. These results also clarify that these beneficial changes occur due to both blood glucose-dependent and –independent mechanisms. The finding that blood glucose *per se* is important since these results suggest that therapeutic strategies to reduce blood glucose levels, regardless of site of action or mechanism(s), in contexts of insulin resistance and type 2 diabetes may ultimately benefit multiple tissues involved in gluco-regulation. Here we focus on improvement to glucose disposal in peripheral tissues benefitting the liver, but the converse remains an area of on-going study. Additional work is also needed to better understand glucose-independent regulatory factors that may be exploited to positively impact hepatic glucose metabolism.
